# Evidence for Quantum Chemical Effects in Receptor-Ligand Binding Between Integrin and Collagen Fragments — A Computational Investigation With an Impact on Tissue Repair, Neurooncolgy and Glycobiology

**DOI:** 10.3389/fmolb.2021.756701

**Published:** 2021-11-16

**Authors:** Thomas Eckert, Jan von Cosel, Benedict Kamps, Hans-Christian Siebert, Ruiyan Zhang, Ning Zhang, Konstantinos Gousias, Athanasios K. Petridis, Dimitrios Kanakis, Konstantin Falahati

**Affiliations:** ^1^ RISCC Research Institute for Scientific Computing and Consulting, Heuchelheim, Germany; ^2^ Institut für Veterinärphysiologie und Biochemie, Fachbereich Veterinärmedizin, Justus-Liebig- Universität Gießen, Gießen, Germany; ^3^ Fachbereich Biologie und Chemie, Hochschule Fresenius University of Applied Sciences, Idstein, Germany; ^4^ RI-B-NT Research Institute of Bioinformatics and Nanotechnology, Kiel, Germany; ^5^ Institute of BioPharmaceutical Research, Liaocheng University, Liaocheng, China; ^6^ Klinik für Neurochirurgie, Klinikum Lünen, St.-Marien-Hospital, Akad. Lehrkrankenhaus der Westf. Wilhelms-Universität Münster, Lünen, Germany; ^7^ Medical School, Heinrich-Heine-Universität Düsseldorf, Düsseldorf, Germany; ^8^ Institute of Pathology, University of Nicosia Medical School, Nicosia, Cyprus

**Keywords:** computational chemistry, computational biophysics, receptor-ligand binding, electronic structure, transition metal chemistry, Jahn-Teller-distortion, neuroblastoma cells, NCAM-beta1 integrin interactions

## Abstract

The collagen-integrin interactions are mediated by the doubly charged Mg^2+^ cation. In nature this cation seems to have the optimal binding strength to stabilize this complex. It is essential that the binding is not too weak so that the complex becomes unstable, however, it is also of importance that the ligand-receptor binding is still labile enough so that the ligand can separate from the receptor in a suited environment. In the case of crystal growing for experimentally useful integrin-collagen fragment complexes it turned out that Co^2+^ cations are ideal mediators to form stable complexes for such experiments. Although, one can argue that Co^2+^ is in this context an artificial cation, however, it is now of special interest to test the impact of this cation in cell-culture experiments focusing on integrin-ligand interactions. In order to examine, in particular, the role cobalt ions we have studied a Co^2+^ based model system using quantum chemical calculations. Thereby, we have shown that hybrid and long-range corrected functional, which are approximations provide already a sufficient level of accuracy. It is of interest to study a potential impact of cations on the binding of collagen-fragments including collagens from various species because different integrins have numerous biological functions (e.g. Integrin – NCAM (Neural cell adhesion molecule) interactions) and are triggered by intact and degraded collagen fragments. Since integrin–carbohydrate interactions play a key role when bio-medical problems such as tumor cell adhesion and virus-host cell infections have to be addressed on a sub-molecular level it is essential to understand the interactions with heavy-metal ions also at the sub-atomic level. Our findings open new routes, especially, in the fields of tissue repair and neuro-oncology for example for cell-culture experiments with different ions. Since Co^2+^ ions seem to bind stronger to integrin than Mg^2+^ ions it should be feasible to exchange these cations in suited tumor tissues although different cations are present in other metalloproteins which are active in such tissues. Various staining methods can be applied to document the interactions of integrins with carbohydrate chains and other target structures. Thereby, it is possible to study a potential impact of these interactions on biological functions. It was therefore necessary to figure out first which histological–glycobiological experimental settings of tumor cells are suited for our purpose. Since the interactions of several metalloproteins (integrin, ADAM12) with polysialic acid and the HNK-1 epitope play a crucial role in tumor tissues selected staining methods are proper tools to obtain essential information about the impact of the metal ions under study.

## Introduction

A detailed understanding of integrin–ligand interactions requires that the impact of ligand structures is considered (Racine-Samson et al., 1997; Gabius et al., 2004; Petridis et al., 2011; Glinskii et al., 2014; Hussein et al., 2015; Cornelissen and Van Vliet, 2016; Marsico et al., 2018; Sharma et al., 2020; Eckert et al., 2021). The corresponding data are necessary to understand cellular mechanisms in neuroblastoma and in glioblastoma cells (Hemler, 1990; André et al., 2005; Bhunia et al., 2010; Petridis et al., 2011; Stötzel et al., 2012; Tsvetkov et al., 2012; Kanakis et al., 2013; Zhang et al., 2016a; Malric et al., 2017; Martinez-Olivera et al., 2018; McCarthy et al., 2018; Van Agthoven et al., 2019). This is possible on a sub-molecular size-level, however, as well as outlined in this article also on a sub-atomic scale where orbital-structures play an essential role. In this context it is important to analyze the influence of the heavy metal ion, which is involved in the binding process. Integrin-ligand interactions are mediated by Mg^2+^.It has been shown that the Mn^2+^ions stabilize integrin-ligand complexes [5FFO (Dong et al., 2017) and 6MSU (Van Agthoven et al., 2019)]. This leads to deeper insights in the structure function relationship of integrin–ligand model structures (Racine-Samson et al., 1997; Van Agthoven et al., 2019). Especially, when the heavy metal ion Co^2+^ is taken into account one can expect interesting results on a fundamental level regarding integrin-ligand interactions. The *α*1*β*1, *α*2*β*1, *α*10*β*1 and α11β1 integrins constitute a subset of the integrin family with affinity for GFOGER-like sequences in collagens. Integrins α1β1 and α2β1 were originally identified on various activated T-cells (Hemler, 1990). These integrins have been expressed on a number of cell types including platelets (α2β1) , vascular cells (α1β1,α2β1) (Racine-Samson et al., 1997), epithelial cells (α1β1,α2β1) (Lee and Streuli, 2014) and fibroblasts (α1β1,α2β1) (Heino and Massagué, 1989; Gailit et al., 1996; Pirilä and Heino, 1996). Potential structural differences of jellyfish collagen, which might influence the integrin-mediated adhesion mechanisms of vertebrate cells on cnidaria collagen are analyzed (Zhang et al., 2019). This probably results from a lack of integrin binding sites and the existence of an alternative binding mechanism such that cells kept their round shape on jellyfish collagen, preventing chondrocytes from de-differentiation. Thus, collagen from e. g. *R. esculentum* is a very suitable and promising material for cartilage tissue engineering (Hoyer et al., 2014).

One has observed Mg^2+^-dependent binding of the α1 I domain to the peptides in the following rank order: III-7 (GLOGEN), II-28 (GFOGER), II-7 and II-8 (GLOGER), II-18 (GAOGER), III-4 (GROGER) (Hamaia et al., 2012). It is important to note that special peptide sequences of collagen are able to influence the differentiation of cells in various ways. Our findings obtained by SPR measurements in combination with NMR and *in silico* calculations (Siebert et al., 2010) provide valuable hints to influence to such differentiation processes just by the choice of various suited cations. In order to understand the related biophysical processes on a deeper level quantum chemical calculations with different ions have to be carried out. We can show that the magnesium and cobalt cations are the most promising and interesting ones in this case (Jahn et al., 1937).

The structural-functional correlations between these ligand-receptor interactions are influencing cell growth and cell differentiation in a characteristic way. The sequence GVOGEA bound weakly to PC12 cells and strongly to activated Rugli cells or to an activated α1 I domain, but not to the α2 I domain or to C2C12 cells expressing α2β1 or α11β1. Thus, GVOGEA is specific for α1β1. Although, recognized by both binding sites α2β1 and α11β1, GLOGEN is a better ligand for binding site α1β1 compared with GFOGER. Finally, using biosensor assays, one can show that although GLOGEN is able to compete for the α1 I domain from collagen IV, GFOGER is much less potent, as shown previously. These data confirm the selectivity of GFOGER for binding site α2β1 and establish GLOGEN as a high affinity for binding site α1β1 for this peptide sequence (Hamaia et al., 2012).

Collagen contains specific cell adhesion domains, including the arginine- glycine-aspartic acid (RGD) motif. After the integrin receptor on the cell surface binds to the RGD motif on the collagen molecule, cell adhesion is actively induced. This interaction contributes to the promotion of cell growth as well as to the differentiation and the regulation of various other cell functions. In the fields of nanomedicine and nanopharmacology the structural–function relationship of biomolecules are described on a sub-molecular level in a clinical context. However, this is also the field of classical structure biology. However, the fields of nanomedicine and nanopharmacology are reaching much far. In the case it is necessary also the sub-atomic size level is included. The theoretical results are of highest importance for clinical research because no pathologist would start with such sophisticated and time-consuming experiments on human brain tissues without expecting diagnostic and therapeutic relevant results after heavy metal ion replacements.

## Materials and Methods

### DFT Calculations

**FIGURE 1:**
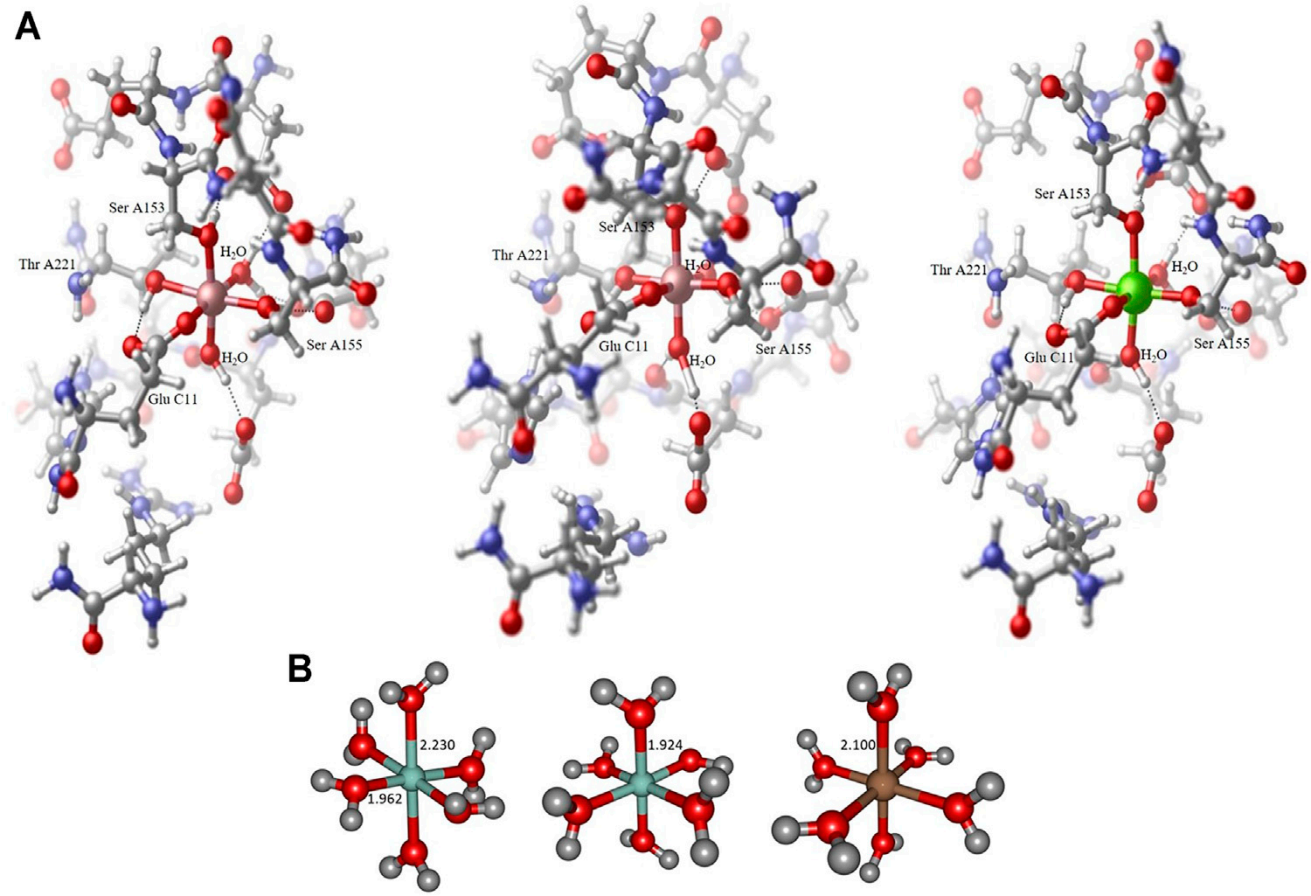
Collagen-integrin model complex containing a Co^2+^ center in a Jahn-Teller distorted octahedral ligand sphere (left), model complex containing a Co^3+^ center in an almost perfect octahedral ligand sphere (center) and model complex containing a Mg^2+^ center in an almost perfect octahedral ligand sphere (right). All structures obtained at the B3LYP/6-31G* level of theory, hydrogen bond interactions to adjacent amino acid residues indicated by dotted lines. The Collagen-integrin model complex contains the amino acids Asp A151, Glu A152, Ser A153, Asn A154, Ser A155, Thr A221, Asp A254, Gly A255, Glu A256, Ser A257, His A258, Arg B12 und Glu C11 and two water molecules of the structure 1DZI (37). The direct ligands on the divalent metal ions are Ser A153, Ser A155, Thr A221, Glu C11 and two water molecules **(A)**. Hexaaqua model complex containing a Co^2+^ center in a Jahn-Teller distorted octahedral ligand sphere of water molecules, axial R_Co−O_ distance 2.230 Å, equatorial R_Co−O_ distances 1.962−1.967 Å (left), hexaaqua model complex containing a Co^3+^ center in an almost perfect octahedral ligand sphere of water molecules, all R_Co−O_ distances 1.924 Å (center) and hexaaqua model complex containing a Mg^2+^ center in an almost perfect octahedral ligand sphere of water molecules, all R_Mg−O_ distances 1.924 Å (right). All structures obtained at the B3LYP/6-31G* level of theory **(B)**.

All geometries considered in this study have been optimized at the long- range corrected CAM-B3LYP (Yanai et al., 2004) and the standard hybrid B3LYP (Becke, 1993) functional level of theory in combination with Ahlrichs SVP (Schäfer et al., 1992) and Pople 6-31G* (Hehre et al., 1972) basis set qualities as implemented in the Gaussian16 program package (Frisch et al., 2016). The B3LYP functional is well-established and tested in the field of transition metal computational chemistry, especially in the context of cobalt-containing systems of different multiplicity (Vargas et al., 2006). All minimum structures have been verified by analysis of the Hessian. Based on the crystal structure (PDB code 1DZI (Emsley et al., 2000) the model systems have been constructed using Hyperchem 8.0 (Hypercube (2007). Hyperch, 2007) to mimic the electronic environment of the cobalt and magnesium complexes respectively. All amino acid residues except the ones indicated in Figure 1A have been cleaved and the ends have been capped with methyl groups. The amino acids (Asp A151, Glu A152, Ser A153, Asn A154, Ser A155, Thr A221, Asp A254, Gly A255, Glu A256, Ser A257, His A258, Arg B12 und Glu C11) and two water molecules were used for the model. All residues are completely free to move during the calculations. Solvation effects have been addressed by means of the PCM (Miertuš et al., 1981; Miertus̃ and Tomasi, 1982) solvation model. In line with literature findings our benchmark calculations have indicated satisfying overall performance of the chosen hybrid and long-range corrected functional quality regarding geometric and electronic features of the metal complexes under consideration.

### MD-Simulation

The protein-complex 1DZI (Emsley et al., 2000) MD-simulation was run with YASARA 20.10.14 (Krieger and Vriend, 2014). The setup included an optimization of the hydrogen bonding network (Krieger et al., 2012) to increase the solute stability, and a pKa prediction to fine-tune the protonation states of protein residues at the chosen pH of 7.4 (Krieger et al., 2006). Na^+^ and Cl^−^ ions were added with a physiological concentration of 0.9%, with an excess of either Na^+^ or Cl^−^ to neutralize the cell. After steepest descent and simulated annealing minimizations to remove clashes, the simulation was run for 100 nanoseconds using the AMBER14 force field (Maier et al., 2015) for the solute, GAFF2 (Wang et al., 2004) and AM1BCC (Jakalian et al., 2002) for ligands, spezial amino acids (hydroxyprolin) and ions (Mg^2+^, Co^2+^, and Co^3+^) and TIP3P for water. The cutoff was set to 8 Å for Van der Waals forces (the default used by AMBER (Hornak et al., 2006), no cutoff was applied to electrostatic forces (using the Particle Mesh Ewald algorithm (Essmann et al., 1995). The equations of motion were integrated with a multiple timestep of 1.25 fs for bonded interactions and 2.5 fs for non-bonded interactions at a temperature of 310 K, 0,993,249 g/ml density and a pressure of 1 atm (NPT ensemble) using algorithms described in detail previously (Krieger and Vriend, 2015). After inspection of the solute RMSD as a function of simulation time, the first 10 ns were considered equilibration time and excluded from further analysis.

Graphical representation of the molecular geometries has been produced with the aid of CylView 1.0b (Legault, 2009) and Chemcraft (Zhurko, 2005).

### Cell Assay Part

The Methods of the Cell assay part are described in the Materials and Methods section of reference (Petridis et al., 2011).

### Explant Cultures of Subventricular Zone

Seven days old mice were sacrificed by decapitation under hypothermic anesthesia. The brains were removed and transferred into serum-free DMEM/F-12 medium supplemented with 1% penicillin/streptomycin (GIBCO-BRL; Carlsbad, CA), and 400 μm thick sagital slices were cut with a vibratome. Circular pieces (about 1 mm^2^) of the subventricular zone region l were extracted and put in DMEM/F-12 medium supplemented 1% penicillin/streptomycin. The cultures were treated with 6 U/ml of a polysialic acid (PSA)-specific endoneuraminidase (endo N). This amount of endo N is sufficient to completely remove PSA from the tissue. Armenian hamster monoclonal anti-β1 (10 μg/ml; BD Biosciences) was added together with endo N to the cultures to assess the role of β1-integrin in endo N-induced neuritogenesis. Untreated cultures were used as controls. All animal care and experimentation were carried out according to MSKCC (Memorial Sloan Kettering Cancer Center) institutional guidelines.

### Cell Line

The cell line used in this study was the human neuroblastoma SH-SY5Y. The cell line was cultured in Dulbecco`s modified medium DMEM/F12 supplemented with 15% heat inactivated fetal calf serum (FCS) and 1% penicillin/streptomycin and Zeocin.

### Immunocytochemistry

Cells were fixed in 4% paraformaldehyde at RT for 10 min, washed in PBS, then incubated with primary antibodies at 37°C for 2 h. After washing with PBS and incubation for 1 h at 37°C with the secondary antibodies, the cells were mounted in mowiol (Calbiochem; San Diego, CA). Primary antibodies used: mouse monoclonal anti-PSA (5A5, 1:1,000), armenian hamster monoclonal anti-β1 Integrin (1:200 BD Biosciences), mouse monoclonal anti-NF-M (145k) antibody for neurofilament staining (Chemicon), mouse monoclonal anti-NCAM (123C3; 1:150; Santa Cruz, Santa Cruz, CA). Secondary antibodies were FITC and Cy3 conjugated (Jackson Laboratories; West Groove, PA). Immunostaining with secondary antibodies alone was used as negative control. Cells were observed under a Zeiss 510LSM confocal microscope.

### Cell Culture of the Human Neuroblastoma Cell Line SH-SY5Y Under Various Too Much Treatments

The PSA-positive neuroblastoma cell line SH-SY5Y (ATCC; Rockville, MD) was plated on glass coverslips and cultivated in Dulbecco’s modified medium DMEM/F12 supplemented with 15% heat inactivated fetal calf serum (FCS) and 1% penicillin/streptomycin. After cells reached a confluency of 60%, all-trans-retinoic acid (RA) (10 μM) was added. Retinoic acid treated and completely untreated cells were used as controls. Four days after addition of retinoic acid, 20 U/ml endo N was added together with serum and antibiotic free DMEM: F12 medium with or without blocking antibodies for 24 h. Antibodies used for blocking were, armenian hamster monoclonal anti-β1 (10 μg/ml; BD Biosciences) and rabbit polyclonal anti-NCAM Fab’-antibody 10 μg/ml (R004).

### Statistical Analysis

All experiments were repeated at least four times in triplicate. Statistical significance was assessed using analysis of variance (ANOVA).

### AFM

The Methods of the AFM are described in the Materials and Methods section of reference (Stötzel et al., 2012).

### Immunohistochemical Experiments with the Antibodies Against ADAM12 and GFAP

As far as the immunohistochemistry experiments with the antibodies against ADAM12 and GFAP in the oligoastrocytoma tissues are concerned, these are described in every detail in the study published by Kanakis et al. (2013). In particular, regarding the quantification of the two antibodies (i.e ADAM12, GFAP) in the two different cell populations (i.e. neoplastic astrocytes, neoplastic oligodendrocytes) this was performed by estimating the number of immunostained cells for each antibody separately, which was then presented as a percentage value in a scale from 0 (no immunostained neoplastic cells) to ++++ (>75 positive neoplastic cells).

## Results

In this study we show how to correlate experimental results obtained from SPR, NMR and X-ray crystallographic analysis with quantum chemical DFT data regarding the transition metal compounds involved. In detail, the occupation of metal-centered orbitals related to the positively charged ions provide the essential key information regarding a direct impact on the structure-function relationship of this important bio-medical ligand-receptor complex. Experimentally obtained results on the impact of different ions on the stability of collagen-fragment-integrin complexes have been derived by Surface Plasmon Resonance (SPR) experiments by some of us (Siebert et al., 2010). However, as outlined in a review article (Zhang et al., 2016b) and in three original research articles (Siebert et al., 2001; Lenthe et al., 2004; Eckert et al., 2012) it is necessary to include ab initio or DFT calculations when an influence of the sub-atomic size level, especially, the impact of orbital shapes has to be analyzed in detail. The cation requirements of integrins (Xiong et al., 2002; Sen et al., 2018) are here studied on such a sub-atomic level and contributes in a significant way to an understanding of the binding processes in such complexes.

The amount of geometric distortion of the metal complex can be typically assigned to the structural strain that is imposed on the central cation by the respective ligand sphere, i.e. in the vicinity of a rather rigid peptide chain backbone the hexacoordinate Co^2+^ tends to depart from the assumed Jahn-Teller distortion where a clear distinction of axial and equatorial ligands is abandoned in favor of the overall complexation (cf. e.g. PDB codes 2GRU (Nango et al., 2008) and (Dowling et al., 2010). This is in sharp contrast to whenever geometric flexibility is increased, e.g. via the incorporation of water molecules as ligands at the complex center, in these cases the skeletal strain is relieved considerably allowing for more appropriate structural parameters for the metal complexation spheres (Chen et al., 2018).

Although the target systems are readily investigated and addressed experimentally, there is no thorough survey of the electronic structure properties documented in literature to date. Therefore, in this work, we aim to determine the electronic structure and geometric properties of selected Co^2+^, Co^3+^, and Mg^2+^ complexes that are chemically sufficiently close to experimentally addressable system at the center of the integrin-collagen complex.

By means of standard DFT methodology, we compare octahedral ligand spheres and Jahn-Teller distortions to indicate the oxidation states of the central cobalt ion thus clarifying the active species in the experimentally crystallized structures. We further characterize the most relevant interaction patterns favoring the complex stability incorporating cobalt and magnesium cations.

The structural analysis of the organometallic core complex is investigated as hexacoordinate model systems that are assumed to sufficiently mimic the experimental conditions. The main electronic influence is expected to arise from the most adjacent substituents that constitute the essentially octahedral ligand sphere of the cobalt and magnesium complexes respectively. As displayed in Figures 1A,B hexaaqua complex as well as a more realistic environment is employed in order to address the electronic structure of the investigated systems. For all cases, the triad of Co^2+^, Co^3+^, and Mg^2+^ central ion is considered constituting a set of 6 target complexes that are evaluated. In our investigation, the hexaaqua complexes serve as natural benchmark systems for the actual system. The structural and electronic properties are evaluated at several levels of theory to ensure best possible and efficient methodology in terms of computational demand and desired chemical accuracy. The influences of hybrid and long-range functionals in particular are crucial for a reliable description of the transition metal center and its nearest surroundings. Furthermore, the basis set dependability of geometric the relevant parameters at the metal center is evaluated.

### Hexaaqua Complexes

As expected, the Co^3+^ and Mg^2+^ hexaaqua complexes are characterized by essentially symmetric octahedral ligand fields according to their electronic configurations while the Co^2+^ exhibits pronounced Jahn-Teller distortion due to its d7 configuration giving rise to a singly occupied $d_{z^{2}}$ orbital. This in turn is reflected by the significant bond elongation of axial ligands to approximately 2.1 Å as depicted in Figure 1B. In contrast, almost identical bond lengths of 1.924 and 2.100 Å are encountered for both axial and equatorial ligands in the Co^3+^ and Mg^2+^ complexes respectively. Table 1 summarizes the bond lengths with respect to the employed level of computational treatment highlighting an essentially method-independent behavior of the geometric parameters. As can be deduced from the numbers, the long-range corrected CAM-B3LYP functional slightly contracts all the bonds by roughly 0.01–0.02 Å while utilization of the Ahlrichs SVP basis set shows mixed trends in comparison to the 6-31G* Pople basis set. Further employment of polarization functions at the hydrogen atoms via the 6-31G** basis set results in slight and systematic overall bond contraction throughout our study which is attributed to the increased flexibility in the description of the electronic structure but does not change the observed trends.

**TABLE 1:** Axial and equatorial bond lengths in Å for the hexaaqua complexes discussed in this study.

Methodology	R_Co−O_^ax^	R_Co−O_^eq^	R_Mg−O_
[Co(OH_2_)_6_]^2+^ complex			
B3LYP/6-31G*	2.239	1.962–1.967	—
CAM-B3LYP/6-31G*	2.204	1.949–1.953	—
B3LYP/SVP	2.189	1.985–1.990	—
CAM-B3LYP/SVP	2.160	1.967–1.974	—
B3LYP/6-31G**	2.236	1.961–1.965	—
CAM-B3LYP/6-31G**	2.200	1.948–1.952	—
B3LYP/6-31G* (PCM)	2.205	1.956–1.958	—
CAM-B3LYP/6-31G* (PCM)	2.102–2.118	1.972–2.031	—
B3LYP/SVP (PCM)	2.186	1.979–1.981	—
CAM-B3LYP/SVP (PCM)	2.068–2.083	1.981–2.079	—
B3LYP/6-31G** (PCM)	2.204	1.954–1956	—
CAM-B3LYP/6-31G** (PCM)	2.172	1.940–1.942	—
[Co(OH_2_)_6_]^3+^ complex			
B3LYP/6-31G*	1.924	1.924	—
CAM-B3LYP/6-31G*	1.903	1.903	—
B3LYP/SVP	1.938	1.938	—
CAM-B3LYP/SVP	1.916	1.916	—
B3LYP/6-31G**	1.922	1.922	—
CAM-B3LYP/6-31G**	1.902	1.902	—
B3LYP/6-31G* (PCM)	1.898	1.898	—
CAM-B3LYP/6-31G* (PCM)	1.881	1.881	—
B3LYP/SVP (PCM)	1.909	1.909	—
CAM-B3LYP/SVP (PCM)	1.888	1.888	—
B3LYP/6-31G** (PCM)	1.894	1.894	—
CAM-B3LYP/6-31G** (PCM)	1.875	1.875	—
[Mg(OH_2_)_6_]^2+^ complex			
B3LYP/6-31G*	—	—	2.100
CAM-B3LYP/6-31G*	—	—	2.081
B3LYP/SVP	—	—	2.089
CAM-B3LYP/SVP	—	—	2.070
B3LYP/6-31G**	—	—	2.099
CAM-B3LYP/6-31G**	—	—	2.081
B3LYP/6-31G* (PCM)	—	—	2.081–2.093
CAM-B3LYP/6-31G* (PCM)	—	—	2.062–2.069
B3LYP/SVP (PCM)	—	—	2.062–2.063
CAM-B3LYP/SVP (PCM)	—	—	2.045
B3LYP/6-31G** (PCM)	—	—	2.078–2.083
CAM-B3LYP/6-31G** (PCM)	—	—	2.059–2.063

As opposed to the cobalt based systems, the magnesium hexaaqua complex is largely stabilized by ion-dipole interactions indicated by the straight geometric orientation of the surrounding water molecules, cf. Figure 1B as opposed to the essentially covalent interactions that dominate the [Co(OH_2_)_6_]^2+^ and [Co(OH_2_)_6_]^3+^ geometries which exhibit bent orientations of the hydrogen atoms in the ligand water molecules with respect to the metal center.

**FIGURE 2:**
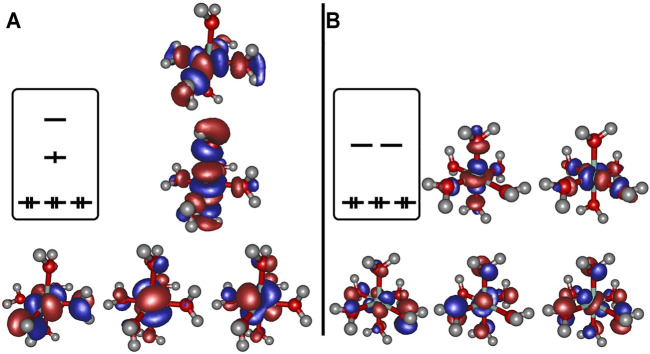
Frontier orbitals obtained at the B3LYP/6-31G* level of theory for [Co(OH_2_)_6_]^2+^ **(panel A)** and [Co(OH_2_)_6_]^3+^ **(panel B)**, orbital occupation sketched for clarity.

Regarding the relevant electronic configurations for the [Co(OH_2_)_6_]^2+^ and [Co(OH_2_)_6_]^3+^ complexes respectively, the typical scheme is obtained in terms of the five d-orbitals of the central cobalt ion that are intermixed with the ligand orbitals as outlined in panels A and B of Figure 2. The resulting orbital occupations patterns can be rationalized as $d_{xy}^{2}d_{xz}^{2}d_{y^{z}}^{2}d_{z^{2}}^{1}d_{x^{2}-y^{2}}^{0}$ for the Co^2+^ and $d_{xy}^{2}d_{xz}^{2}d_{yz}^{2}d_{z^{2}}^{0}d_{x^{2}-y^{2}}^{0}$ for the Co^3+^ systems respectively, perfectly in line with the expected geometries for distorted and undistorted d7 and d6 ligand fields.

**FIGURE 3:**
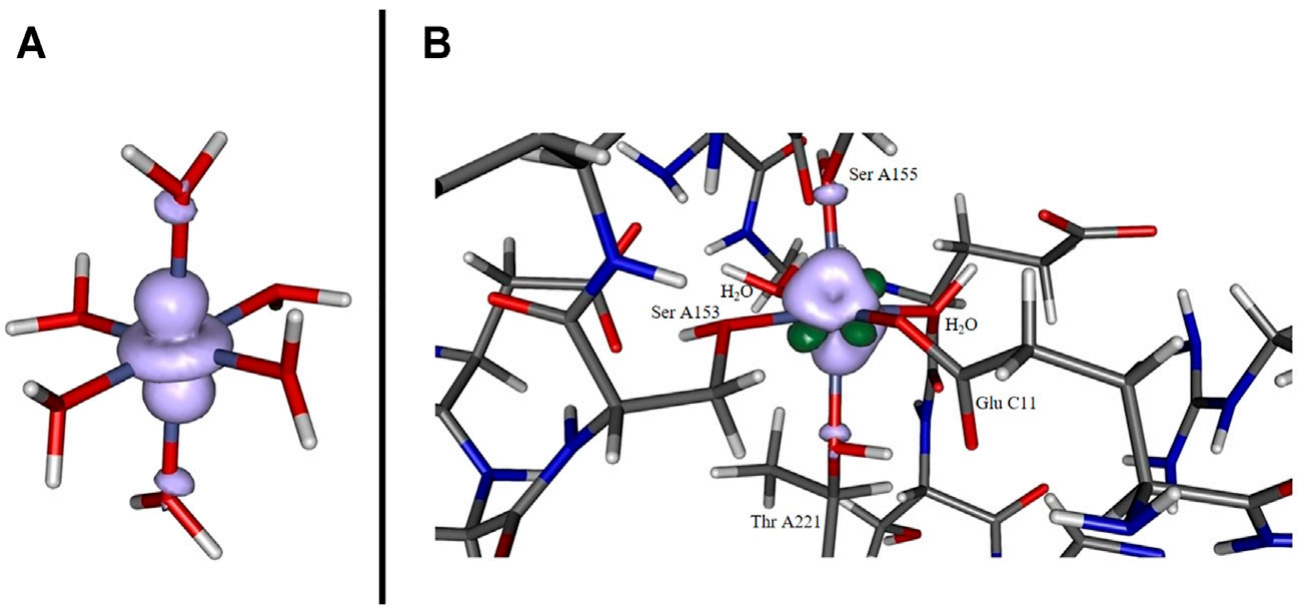
Spin density for [Co(OH_2_)_6_]^2+^ **(panel A)** and for the cobaltous model system **(panel B)** computed at the B3LYP/6-31G* level of theory. In the former case, a pronounced localization at the metal site originating from the singly occupied d_z_2 orbital. In the latter case the distribution is more diffuse and additionally features significant spin polarization contributions (dark green lobes).

The depiction of the net spin density (cf. panel A of Figure 3) emphasizes the notion of the singly occupied $d_{z}2$ -orbital strongly localized at the cobalt site, a result that is anticipated given the allowed mixing pattern in an octahedral coordination sphere for the set of $e_{g}$ orbitals at the cobalt center and the donor orbitals (that stem from the oxygen in the axial water ligands) that constitute σ -type interactions. Regarding the overall positive sign of the spin density, the predominance of spin delocalization to the adjacent ligands is apparent at the chosen cutoff in Figure 3.

**FIGURE 4:**
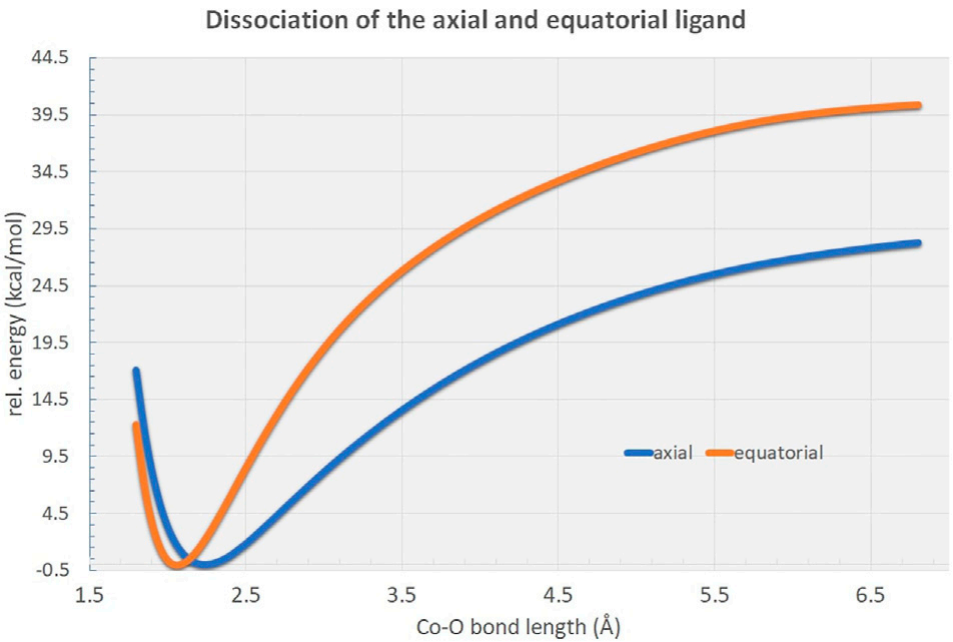
Rigid potential energy surface scans along the R_Co−O_ distance in the [Co(OH_2_)_6_]^2+^ hexaaqua complex at the B3LYP/6-31G* level of theory. As can be figured from the curves, the axial ligand dissociation is energetically highly favored in comparison to the equatorial ligands by approximately 12 kcal/mol.

Potential energy surface scans reveal distinct energetic differences in bond dissociation curves for axial and equatorial ligand cleavage from the [Co(OH_2_)_6_]^2+^ complex (Figure 4). Due to the elongated bond distances at the axial positions the net dissociation energy is expected to be considerably lowered by approximately 12 kcal/mol indicative of possible biological relevance in the Co^2+^-mediated processes in the full protein environment.

### Integrin-Collagen Model Complex

Our integrin-collagen model system consists of an inner layer of electronic interaction patterns inspired by the original chemical site at the crystal structure from the PDB code 1DZI by Emsley et al. (2000). As can be deduced only two coordinating water molecules are directly surrounding the central ion while the side chain residues of the protein chain occupy the four remaining positions in the hexacoordinate ligand sphere. The incorporation of the metal cation in the ligand sphere of the protein background affects the geometric arrangement of the complex and slightly alters the electronic interactions. Most prominently, the complex symmetry is lowered to certain degrees as the various bond lengths are determined by the nature and electronic properties of the respective ligand that is either a water substituent or an amino acid type residue. Table 2 displays the relevant bond lengths for the three investigated collagen-integrin model complexes. While the d7 configuration in case of the cobaltous system still dominates the Jahn-Teller type distorted structural motif, the geometries of the Co^3+^ and Mg^2+^ complexes effectively adapt to the heterogeneous coordination spheres including the isolated water molecules. The cobaltic system for instance consequently shrinks the $R_{Co-O}$ distances facing the oxygen donor atom of the water substituents with respect to the oxygen atoms originating from the amino acids in the geometric proximity. This matter, however, correlates well with the notion of flexible movement of disjoint water ligands in contrast to the rigid backbone of the protein chain. In case of the magnesium system, the overall $R_{Mg-O}$ distances are effectively larger thus facilitating the structural adjustments of the complex given the steric and geometric demands of the respective ligand framework. The inclusion of solvation effects by means of the PCM model (for a water environment) does not alter the overall picture conducting only minimal changes in the bond lengths. In general, the bond contraction originating from the long-range corrected functional seems to outnumber the influence of the continuum model. Therefore, the already compact structure of the model complex does not seem to significantly benefit from additional solvation effects during geometry optimization relying on the PCM approach.

**TABLE 2:** Axial and equatorial bond lengths in Å for the collagen-integrin model complexes discussed in this study.

Methodology	R_Co-O_^ax^(1)	R_Co-O_^ax^(2)	R_Co-O_^eq^(1)	R_Co-O_^eq^(2)	R_Co-O_^eq^(3)	R_Co-O_^eq^(4)
	Thr A221	Ser A155	Glu C11	H_2_O 306	H_2_O 307	Ser A153
Co^2+^ complex						
B3LYP/6- 31G*	2.303	2.177	1.952	1.975	2.001	2.073
CAM-B3LYP/6- 31G*	2.304	2.190	1.913	1.931	1.940	2.004
B3LYP/6-31G* (PCM)	2.395	2.263	1.913	1.945	1.956	2.013
CAM-B3LYP/6-31G* (PCM)	2.323	2.209	1.905	1.936	1.940	2.001
Co^3+^ Complex						
B3LYP/6- 31G*	1.842	1.883	1.915	1.925	1.974	1.991
CAM-B3LYP/6- 31G*	1.825	1.866	1.896	1.898	1.945	1.962
B3LYP/6-31G* (PCM)	1.839	1.896	1.908	1.933	1.979	1.983
CAM-B3LYP/6-31G* (PCM)	1.822	1.877	1.891	1.907	1.951	1.953
Mg^2+^ Complex						
B3LYP/6- 31G*	2.051	2.053	2.066	2.116	2.209	2.214
CAM-B3LYP/6- 31G*	2.035	2.036	2.046	2.088	2.174	2.183
B3LYP/6-31G* (PCM)	2.045	2.063	2.065	2.132	2.203	2.208
CAM-B3LYP/6-31G* (PCM)	2.031	2.046	2.046	2.105	2.170	2.180
Exp. (38)	2.459	2.376	2.311	2.259	2.218	2.212

**FIGURE 5:**
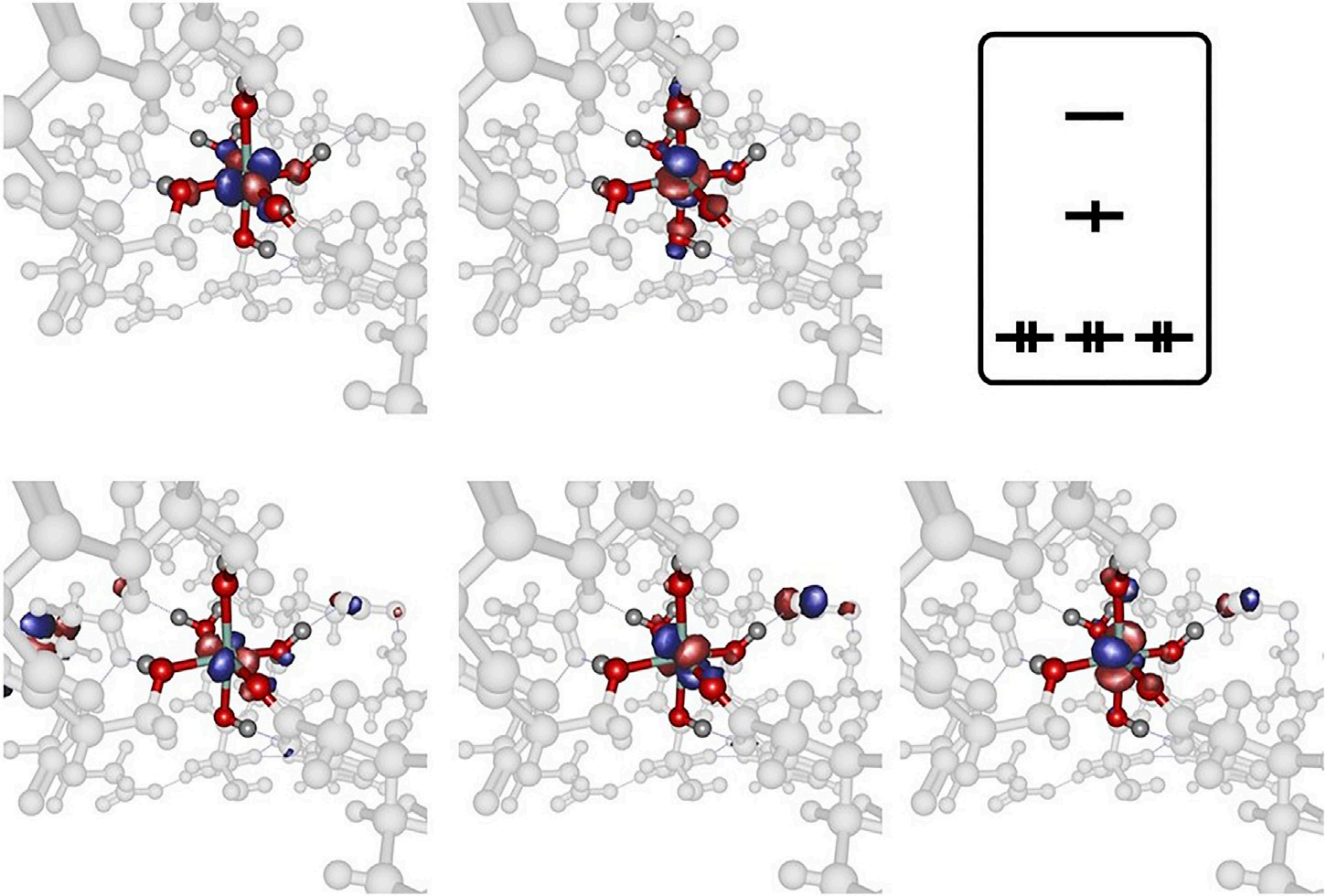
Frontier orbitals for the Co^2+^ model complex obtained at the B3LYP/6-31G* level of theory and sketched population scheme. Only orbitals with significant Co contribution are displayed. While the essential shape of the five Co d-orbitals is conserved, substantial deformation and bending of the respective orbital orientation is apparent. The amino acid residues are shaded collectively in gray color for the sake of clarity.

Analysis of the three model cases does, however, demonstrates the general tendency to asymmetrize the complex from its either perfect octahedral or Jahn-Teller distorted shape into somewhat tilted hybrid structures that qualitatively feature two subsets of bond lengths. This finding raises the doubt for a distinct cobaltous character of the central ion in the embedding of the experimental system. In Figure 5 the frontier orbital occupation (with significant Co contribution) are depicted for the cobaltous system. As can be deduced, the orbital shape and orientation is clearly affected by the asymmetric electronic environment due to the amino acid residues in direct proximity of the metal center. Thus, the tilting and bending of orbital character is apparent to optimize the interactions upon protein embedding of the cation unlike in the hexaaqua system where orbital deformation is less distinct (cf. panel A of Figure 2).

The spin density distribution in the Co^2+^ model complex (cf. panel B of Figure 3) is likewise altered in comparison to the hexaaqua ligand sphere: while the spin delocalization seems to dominate the situation in a coordination sphere of six water molecules as stated above, the asymmetric surroundings as well as the bond elongation with respect to the paramagnetic center induce a more complex spin density distribution. For instance, the additional dark green lobes are indicative of substantial spin polarization phenomena in the vicinity of the Co center. Given the more involved interaction patterns the Mulliken spin density of the cobalt center decreases ever so slightly from approximately 0.966 in the hexaaqua system to 0.961 in the model system based on our computations at the B3LYP/6-31G* level of theory. This trend, however, depends crucially on the quality of the long-range interaction description as provided by the CAM-B3LYP functional. In the latter case the numbers change from 0.962 to 0.971 thus showing a reversed picture with a more isolated spin density accumulated at the metal center. The effect of PCM solvation further intensifies the spin density aggregation at the Co center. The numbers are inferred from Table 3. As the computation of the spin density is, in general, less sensitive with respect to the employed level of theory and basis set quality (Cano et al., 1998; Ruiz et al., 2005; Giner and Angeli, 2016; Philips et al., 2010) the overall method dependency is, however, comparably negligible.

**TABLE 3:** Mulliken spin densities at the cobaltous center of the hexaaqua and the model complex at different levels of theory.

Methodology	[Co(OH_2_)_6_]^2+^ complex	Co^2+^ model complex
B3LYP/6-31G*	0.965782	0.960802
B3LYP/6-31G*(PCM)	0.958735	0.982862
CAM-B3LYP/6-31G*	0.961517	0.970751

**FIGURE 6:**
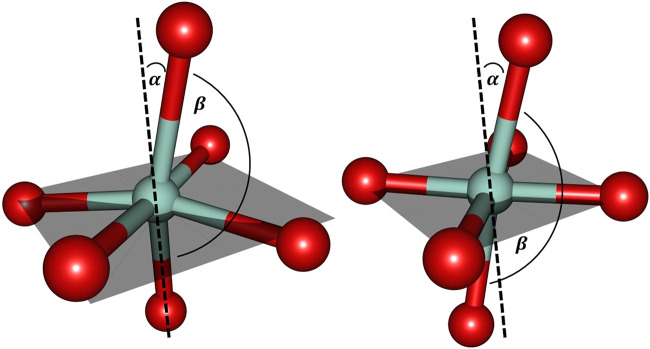
Cobalt coordination sphere extracted from the crystal structure data by Emsley et al. (2000) (left) and the ligand sphere for the freely optimized [Co(OH_2_)_6_]^2+^ complex (right) using the B3LYP/6-31G* level of theory. For the sake of clarity only the directly bound oxygen atoms are displayed. The gray shaded plane indicates the arrangement of equatorial ligands: in the optimized hexaaqua complex, the equatorial oxygen atoms essentially span a plane with minimal Co out-of-plane deviation while the experimental system features pronounced deformations. α denotes the deviation relative to the plane normal and β_O−Co−O_ depicts the axial angle of the complex. The values for the angles are approximately given as: α = 15° and β_O−Co−O_ = 165° (left) and α = 10° and β_O−Co−O_ = 173° (right).

Further inspection reveals the marked deviations from the predicted structural arrangement: as Figure 6 suggests, the experimental cobalt complex extracted from the PDB file provided by Emsley et al. (2000) lacks the intrinsic symmetric properties that are characteristic for the [Co(OH_2_)_6_]^2+^ system in several ways. Firstly, the pseudo-equatorial ligand atom positions lead to a substantial cobalt out-of-plane relocation (featuring a dihedral angle of approx. 6° with respect the plane) in contrast to the hexaaqua complex. Secondly, the angle of the axial ligands (denoted as $\beta_{O-Co-O}$ in Figure 6) is significantly readjusted from essentially linear to a tilted orientation of approximately 165°. The angle contraction hence correlates with the attenuated distinction of axial and equatorial bond lengths as already illustrated before.

Comparison to the biological magnesium complex is reasonably reflected in the cobalt complex since it similarly allows for geometric rearrangements that are present in the more flexible embedding of the metal ion in an augmented hexacoordinate ligand sphere with elongated bonds as compared to the hexaaqua situation.

### MD Simulations and Verification of the Model System

**FIGURE 7:**
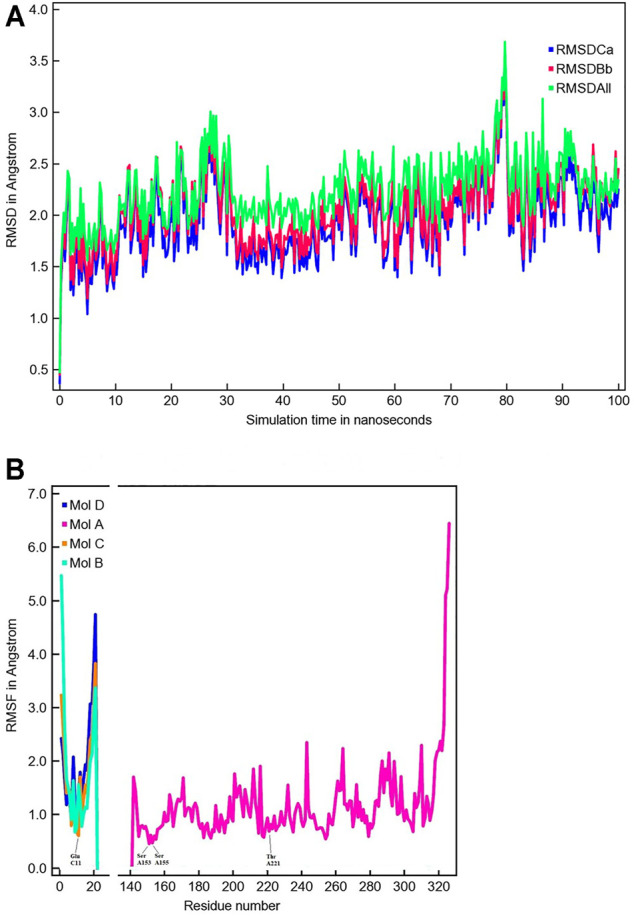
The time evolution of the root mean square deviation (RMSD) measured from the corresponding starting structure 1DZI (Emsley et al., 2000). The different RMSD plots have the following meaning: RMSDCa (blue) is the RMSD for C-alpha, RMSDBb (red) is the RMSD for the backbone and RMSDAll (green) is the RMSD plot for all-heavy atoms **(A)**. The root mean square fluctuation (RMSF) in Angstrom per solute protein residue calculated from the average RMSF of the atoms constituting the residue: integrin Mol A (magenta), collagen Mol B (cyan), collagen Mol C (orange) and collagen Mol D (blue). A RMSF of exactly zero means that that residue number is not present in the Molecule **(B)**.

**FIGURE 8:**
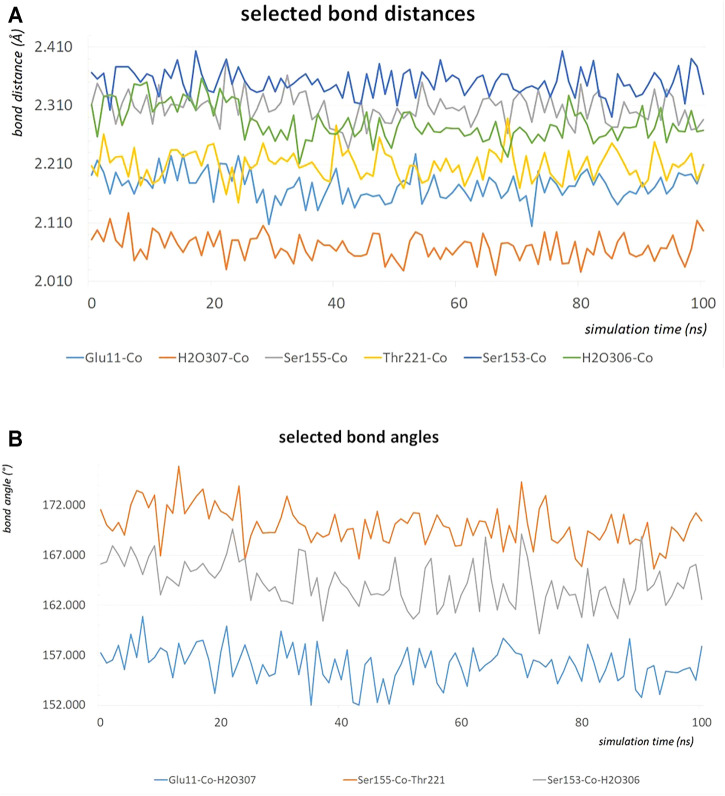
The bond distances in Å of the amino acids Ser A153, Ser A155, Thr A221, Glu C11, water 306 and water307 around the Co^2+^ during an MD simulation of 100 ns **(A)**. The bond angles in degrees of Glu C11-Co^2+^-water 307, Ser A155-Co^2+^-Thr A221 and Ser A153-Co^2+^-water 306 during an MD simulation of 100 ns **(B)**.

The MD simulation of 1DZI (Emsley et al., 2000) has been carried out to assess the structural stability of the protein complex complementary to our DFT analysis of the transition metal center. Our findings are in line with similar MD studies by (Siebert et al., 2010; Zheng et al., 2018) The explicitly mapped coordination sphere of the cobalt ion in our fully quantum mechanical structure turns out to be well-behaved during our optimization and no expansion or compression of our model system is observed indicative of unbalanced estimation of interaction patterns induced by the chemical environment in the native complex. To analyze the MD simulation, the RMSD was determined as a function of 100 ns runtime (Figure 7A). Figure 7A shows an average RMSD of about 2 Å. Higher RMSD values are only present within the range of 27 and 79 ns. In order to determine whether higher RMSD values affect the environment of the Co^2+^ ion, the Co-ligand bond distances have been included for these times in Table 4, displaying overall good agreement with the other evaluated bond distances. To analyze where the structural fluctuations come from, the RMSF values for the individual residuals were calculated (Figure 7B). In particular, the ends of the proteins are shown to be very flexible, with the exception of the N-terminal end of the integrin, which is well stabilized by two neighboring beta sheet structures. In particular, the ends of the proteins are shown to be very flexible, with the exception of the N-terminal end of the integrin (Mol A magenta), which is well stabilized by two neighboring β-sheet structures. The amino acids Ser A153, Ser A155, Thr A221, and Glu C11 exhibit a significantly reduced flexibility as integral parts of the transition metals coordination sphere. During the simulation over 100 ns, the bond distances of the above-mentioned amino acids and two water molecules fluctuated by approx. 0.1 Å and the bond angles by approx. 10 degrees around their equilibrium position (Figures 8A,B). The average bond distances extracted from our MD simulations (up to 100 ns) the complex based on the 1DZI code from the PDB displays satisfactory agreement with our findings based on the density functional treatment of the model system for all levels of theory employed. Incidentally, the asymmetrical coordination sphere of the metal center featuring six gradually different interatomic distances rather than a perfect (Jahn-Teller-based) octahedral geometry is reproduced in both computational approaches. Our integrin-collagen model system consists of an inner layer of electronic interaction patterns inspired by the original chemical site at the crystal structure from the PDB code 1DZI by Emsley et al. (2000).

**TABLE 4:** MD-Simulation of 1DZI (Emsley et al., 2000) with cobalt-complex in Å.

1DZI-Co complex						
Time	Glu 11	H_2_O 307	Ser 155	Thr 221	Ser 153	H_2_O 306
0 ns	2.218	2.040	2.311	2.212	2.376	2.281
10 ns	2.169	2.101	2.279	2.177	2.360	2.300
20 ns	2.146	2.070	2.305	2.245	2.333	2.293
27 ns	2.144	2.084	2.328	2.240	2.333	2.283
30 ns	2.156	2.050	2.304	2.211	2.334	2.250
40 ns	2.203	2.062	2.268	2.277	2.346	2.262
50 ns	2.172	2.043	2.306	2.192	2.310	2.274
60 ns	2.176	2.069	2.302	2.209	2.356	2.271
70 ns	2.148	2.046	2.333	2.169	2.333	2.273
79 ns	2.173	2.078	2.264	2.184	2.323	2.293
80 ns	2.192	2.056	2.347	2.202	2.376	2.298
90 ns	2.179	2.057	2.292	2.172	2.308	2.310
100 ns	2.210	2.097	2.287	2.208	2.330	2.268
average	2.176	2.066	2.302	2.208	2.340	2.281

### Cell Assay Part: Consequences for Cell Experiments in the Field of Neurooncology

**FIGURE 9:**
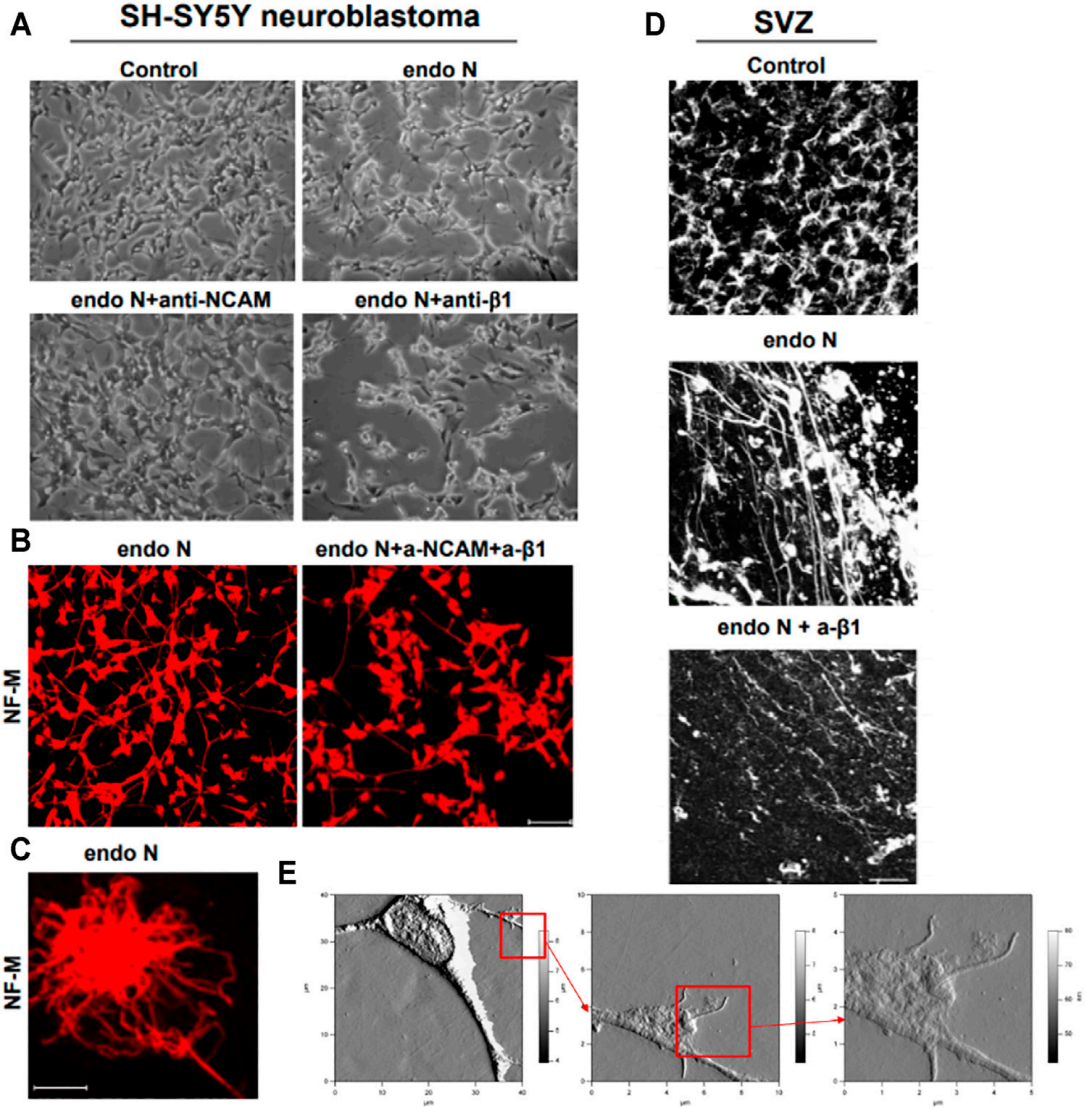
Neuroblastoma cells of a SH-SY5Y cell-line treated with endo N, which specifically cleaves polysialic acid (PSA) from NCAM. Neuritogenesis of these cells occurred **(A)**. Induction of neurofilament M (NF-M)-positive neuronal processes **(B,C)** is clearly inhibited by the use of an NCAM-blocking antibody **(A)**. β1-Integrinblocking antibody significantly inhibited the endo N induced effect on neuritogenesis **(A)**. Inhibition of endo N-induced neuritogenesis by β1-integrinblockage is not only applied in neuroblastoma cells but also in sub ventricular zone (SVZ) progenitor cells. 9D shows that neurite outgrowth in SVZ explant cultures is induced by endo N. Addition of β1-integrin blocking antibody reduces the effect of endo N. Beside polysialic acid (PSA) a sulfated oligosaccharide (the HNK1 epitope) plays an important role as marker on neuroblastoma cells. Therefore, AFM (Atomic Force Microscopy) presentations of a HNK-1 positive neuroblastoma cell are shown **(E**) in different enlargements.

When SH-SY5Y neuroblastoma cells were treated with endo N, which specifically cleaves polysialic acid (PSA) from NCAM, neuritogenesis of these cells occurred (Figure 9A). Induction of neuronal processes, which were neurofilament M (NF-M)-positive (Figures 9B,C), was significantly inhibited by the use of an NCAM-blocking antibody (Figure 9A). Surprisingly, β1-integrin blocking antibody significantly inhibited the endo N induced effect on neuritogenesis (Figure 9A), but not at the same scale as anti-NCAM. Inhibition of endo N-induced neuritogenesis by β1-integrin blockage were not only applied in neuroblastoma cells but also in subventricular zone (SVZ) progenitor cells. Figure 9D shows that neurite outgrowth in SVZ explant cultures is induced by endo N and that addition of β1-integrin blocking antibody reduces the effect of endo N.

**FIGURE 10:**
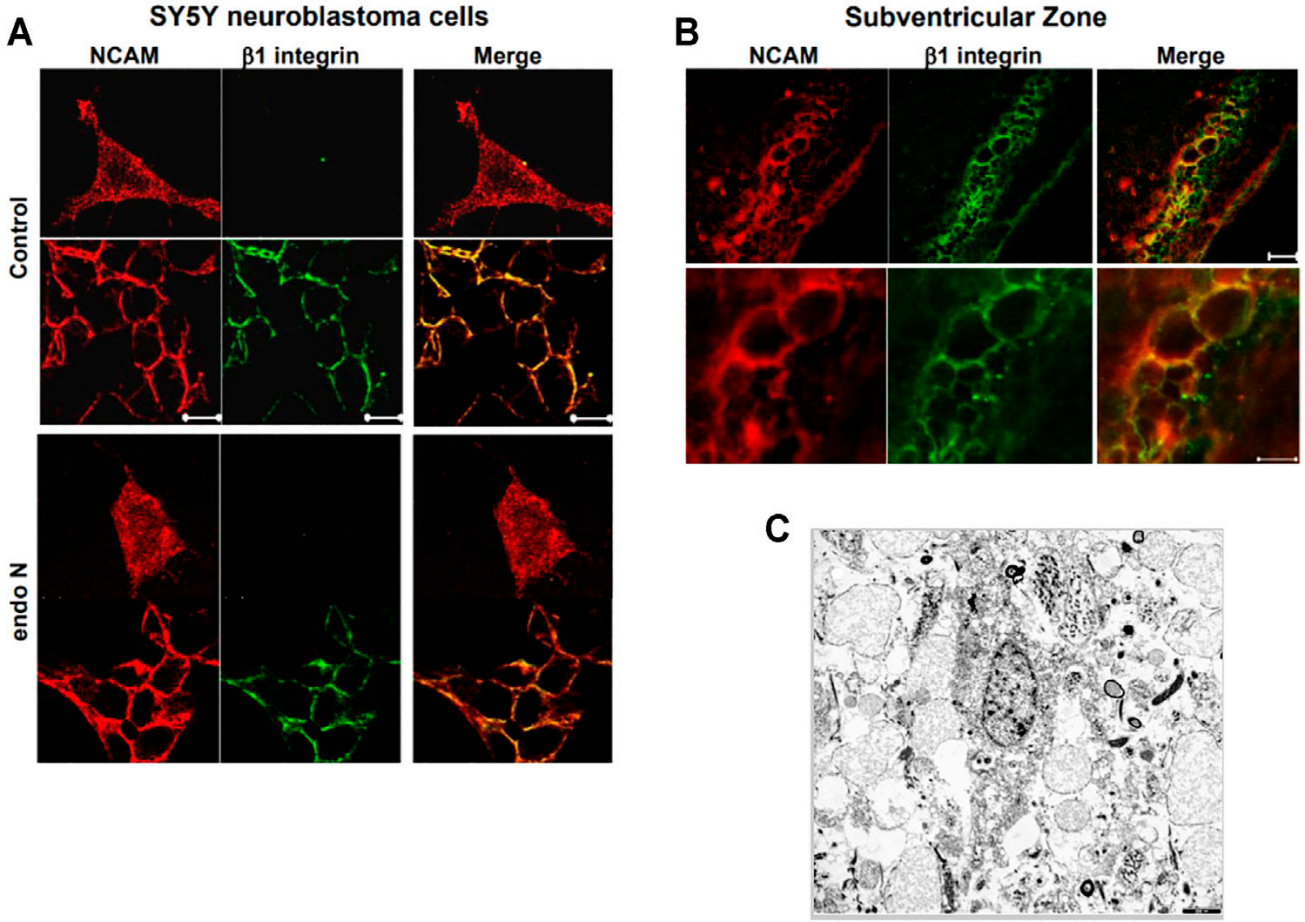
The relation between NCAM and β1-integrinin neuroblastoma cells was performed by an immune staining for both molecules. In the absence and presence of endo N, NCAM colocalized with β1-integrin **(A)**. This colocalization was present at regions where no cell–cell contact occurred and was independent of the presence of endo N **(B)**. β1-integrin was not expressed in isolated cells, whereas NCAM was expressed, but was present all over the cell. In the case of cell–cell contacts, NCAM was localized on the cell surface and β1-integrinwas expressed in contact areas between the cells, as indicated in **(B)** for the sub ventricular zone (SVZ). **(C)** shows cells of a glioblastoma multiform WHO grade 4 (9). In these kind of tumors specific integrin-interactions are also of highest importance.

Our first approach for evaluating the relation between NCAM and β1-integrin in neuroblastoma cells was to immunostain cells for both molecules. We found that in the absence and presence of endo N, some NCAM colocalized with β1-integrin (Figure 10A). This colocalization was present at points where there was cell–cell contact and was independent of the presence of endo N (Figure 10B). β1-integrin was not expressed in isolated cells, whereas NCAM was expressed and was present on cell surface. In the case of cell–cell contact, NCAM was localized on the cell surface and β1-integrin was expressed in contact areas between the cells (Figures 9A, 10B).

For such cell systems it makes sense to carry out experiments with Co^2+^, Mg^2+^ and Mn^2+^ as well as Co^3+^ ions in order to identify the bio-medical role of heavy metal cations on a cell-tissue level.

The cell biological/histological data presented in this study avenues for further experimental settings in which the theoretical molecular modeling results are tested on tumor tissues obtained from neuroblastomas, oligodendrogliomas (WHO grade II-III), glioblastomas (WHO grade IV) and various metastatic cells.

It is known that cell differentiation and migration as well as tissue repair processes strongly depend on the occurrence of polysialic acids and the HNK-1 epitope which are contact-structures of the corresponding cell surfaces. The concentration of the HNK-1 epitope and the length of the polysialic acid chain on a tumor cell surface are suitable prognostic markers to determine the malignancy of such cells. Integrins which are glycoproteins and major players in cell differentiation as well as cell migration processes interact in various ways with the HNK-1 epitope and polysialic acids, thereby, influencing the fate of the cell. A number of questions are still unanswered in respect to this glycobiological processes. Especially, concerning the interaction processes on a sub-molecular level it is essential to evaluate potential tumor cell experiments in relation to the new insights provided by our DFT results. As shown already with SPR experiments it is possible to replace the Mg^2+^ cations of integrin by Co^2+^ cations. In the same way this can be performed for the integrins in tumor cell tissues. According to our molecular modeling studies in which parts of the metalloprotein integrin are analyzed even on sub-atomic size scale it has now tested by staining techniques whether there is an impact on the cells and tissues. This is of particular interest because potential therapeutic options with cobalt salts in cancer research could be a promising option and provide at least new insight in the structure–function relationship of tumor spread and migration. The answer why nature has chosen Mg^2+^ instead of Co^2+^ could then even be answered.

In histology, the glial fibrillary acidic protein (GFAP stain) is carried out to determine whether cells contain the glial fibrillary acidic protein, a protein found in glial cells (Kanakis et al., 2013). The GFAP staining is useful for determining whether a tumor of glial origin. ADAM (A Disintegrin And metalloprotease) 12 has also been implicated in the development of pathogen various forms of cancer, hypertension, liver fibrogenesis, and asthma (Estrella et al., 2009). Since collagen–integrin (Siebert et al., 2010) and collagen–ADAM interactions (Eckert et al., 2021) play a dominant role in cell experiments (Petridis et al., 2011; Martinez-Olivera et al., 2018) where Mg^2+^ is replaced by Co^2+^ we have already discussed their feasibility here.

**FIGURE 11:**
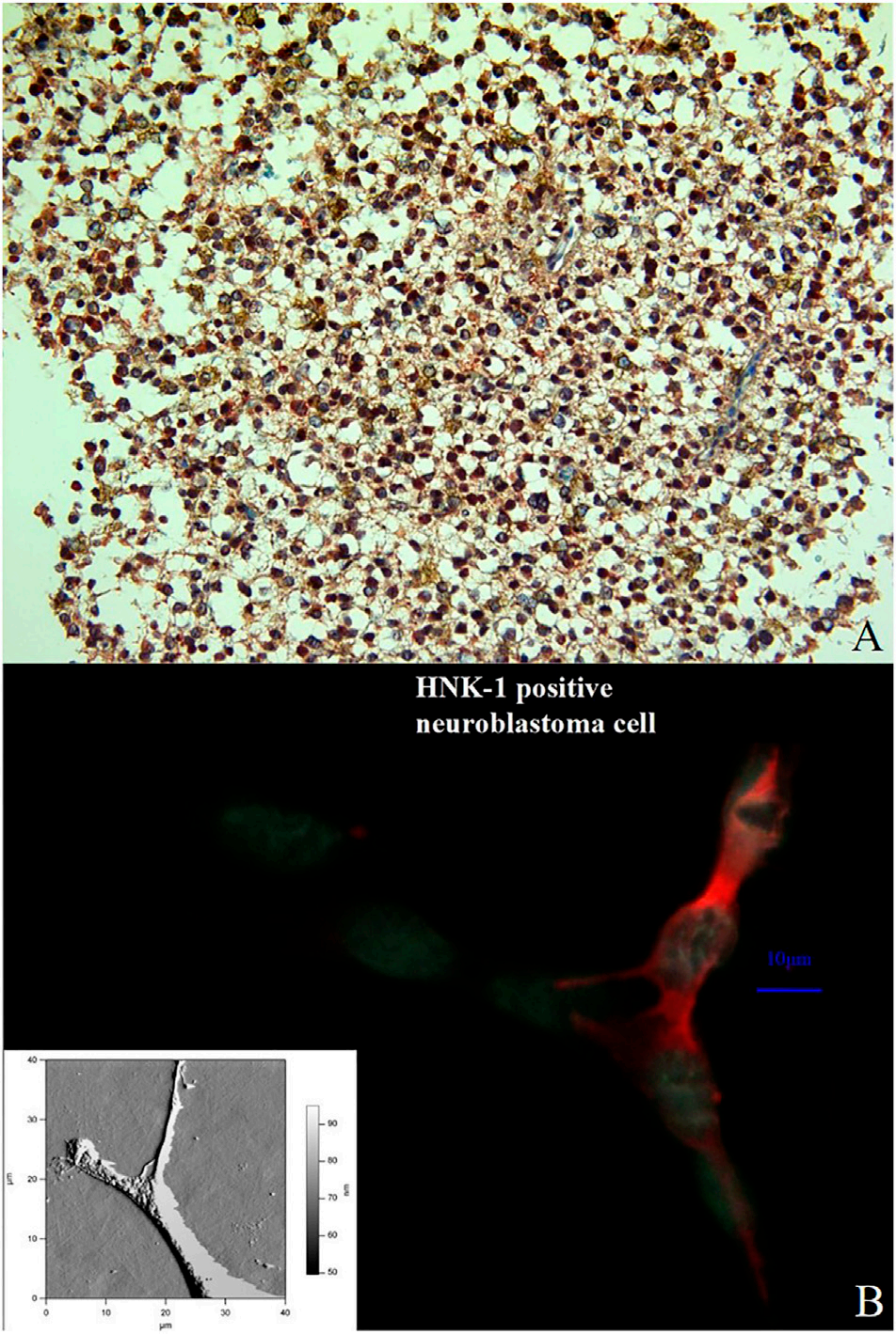
Double immunostaining with the antibodies against ADAM12 (red color) and GFAP (brownish color) in a previously diagnosed oligoastrocytoma WHO grade II. Immuno-histochemistry for these markers identifies and labels two distinct though intermixed tumor-cell populations; the oligodendroglioma cells stain positive for ADAM12 whereas the astrocytoma cells for GFAP. (Magnification × 200) **(A)**. Comparison between an immune stained neuroblastoma cell (the HNK-1 epitope is stained in red) and an AFM presentation of a neuroblatoma cell of the same cell line (lower left corner). The relation between NCAM and β1-integrinin neuroblastoma cells was performed by an immune staining for both molecules **(B)**.

Replacing the Mg^2+^ by a Co^2+^ cation in an integrin it is less complicated than the replacement of such cation in a complete tumor cell tissue probe which is necessary to study a potential impact on cell differentiation and migration processes. Special staining methods which are introduced in Figures 11A,B as well as in Figures 9, 10 can also be used to document the effects of these cation replacements. Nevertheless, one has to keep in mind that in a tumor cell tissue also other proteins with cations are triggering cellular processes (Kanakis et al., 2013). Figure 11A show cells of a previously diagnosed oligoastrocytoma WHO grade II. A vast majority of cells mainly from the oligodendroglioma component expresses the metalloprotease ADAM 12 which interacts with integrins in a specific way (Estrella et al., 2009; Kanakis et al., 2013). The results of our molecular model calculations clearly show that, especially, with integrins, a clear effect can be expected through the replacement of the Mg^2+^ ions by the Co^2+^ ions.

## Discussion

In this study we analyzed the fundamental electronic structure of a Co^2+^ containing integrin-collagen-fragment complex. This complex served as appropriate model system for biologically relevant integrin-collagen structures featuring Mg^2+^ cations at their center. Based on our computational benchmark at the hexaaqua complexes of Co^2+^, Co^3+^ and Mg^2+^ we identify the combination of hybrid and long-range corrected density functional theory with standard basis set sizes of Pople and Ahlrichs quality to be a useful tool for geometry and electronic structure determination in these hexacoordinate transition metal ligand spheres.

In light of our investigations we determined a pronounced Jahn-Teller distortion of the [Co(OH_2_)_6_]^2+^ hexaaqua complex as well as a comparable distortion in our Co^2+^ model system that features the biologically relevant amino acid residues in the ligand sphere of the metal center. This is in line with the general picture of orbital energetic rearrangement due to the singly occupied $d_{z^{2}}$ orbital at the Co^2+^ site. The evaluation of the spin densities further corroborates the notion of a confined net spin localization at the Co^2+^ center. This is in sharp contrast to the [Co(OH_2_)_6_]^3+^ and [Mg(OH_2_)_6_]^2+^ hexaaqua complexes and the Co^3+^ and Mg^2+^ based model systems where a more symmetrically shaped octahedral cavity is adopted in the center of the ligand field.

Our model system that mimics the electronic environment of the collagen-integrin complex has proven to be suited for the evaluation of the effective interaction patterns that are induced by the surrounding amino acid side chains that shape the ligand sphere of the respective central cation, i.e. Co^2+^, Co^3+^ and Mg^2+^. Unlike in the flexible hexaaqua ligand field, the Co^2+^ center in the model system is pinched in a considerably distorted electronic environment that significantly affects the geometric conditions compared to a typical Jahn-Teller-distorted arrangement for d^7^ metals. The clear distinction between elongated axial and considerably shorter equatorial bond distances is partly relieved in favor of a more erratic distribution of geometric parameters such as bond lengths and angles. In our MD simulation, bond lengths and bond angles were also found to be very stable (Figures 8A,B). In view of our MD simulations, we do confidently assume that given the overall structural conservation of the protein complex during our simulations, the transition metal center is crucial to the skeletal integrity of the protein and is therefore adequately described by our model system at the DFT level of theory. Upcoming research aims to combine the theoretical findings in this study in terms of hybrid quantum-classical QM/MM approaches to assess quantitative treatment of the system.

We conclude that consideration of quantum chemical effects for the transition metal complexes discussed in this study is crucial in order to adequately describe the geometry and electronic environments of the structures that are a sensitive function of the electronic configuration of the central cation. In particular, we emphasize the fact that the short interatomic ligand-metal distances in the cobalt based systems indicate a partial covalent bond character in contrast to the magnesium-based complex. It is therefore likely, that the former might be prone to sensitive changes in their biochemical functionality with respect to the oxidation state of the cobalt center due to elongation and compression of axial ligand distances from the center associated with Jahn-Teller type distortions. A clear biological application has not yet been found, but one can imagine that a change in the oxidation state or a change in the spatial direction of the Jahn-Teller distortion results in a change in the bond strength at the cobalt cation in a certain direction and so on perhaps a previously absorbed ligand can be released again at a later point in time. It could be some kind of biological switch.

The studied integrin-collagen complex is stabilized by a Co^2+^ cation which exhibits substantial Jahn-Teller distortion in its crystal structure (Emsley et al., 2000) as well as in our computational calculations discussed here. Depending on the orientation of the axial bond, ligands bind weaker or stronger to the central metal ion. Our analysis shows that bond cleavage of axial and equatorial ligands can vary within a few kcal/mol in binding energy. The quantum chemical DFT data may contribute to a better understanding of the function relationship of integrin ligand complexes including the role of integrins in tissue repair, glycobiology and neurooncology (Racine-Samson et al., 1997; Gabius et al., 2004; André et al., 2005; Bhunia et al., 2010; Petridis et al., 2011; Stötzel et al., 2012; Tsvetkov et al., 2012; Kanakis et al., 2013; Glinskii et al., 2014; Hussein et al., 2015; Zhang et al., 2016a; Cornelissen and Van Vliet, 2016; Malric et al., 2017; Marsico et al., 2018; Martinez-Olivera et al., 2018; McCarthy et al., 2018; Sharma et al., 2020; Eckert et al., 2021). The polysialylated neural cell adhesion molecule (NCAM) as well as the sulfated HNK-1 epitope are together with β1-integrin involved in cell differentiation processes. This includes with changes in the expression of these molecules correlated with changes in the malignancy of tumor cells. Functional correlation between NCAM, HNK-1, collagen, ADAM12 and β1-integrin adhesion and also neurite outgrowth in brain-tumor cells are key interactions in various tissues and can in principle be triggered by the use of certain heavy metal ions. Possible follow-up studies will focus on identification of related three-dimensional structures that exhibit similar Jahn-Teller distortion patterns in order to further elucidate the evidence concerning the relevance of quantum chemical effects in the characteristic mechanism of ions in protein complexes. Thereby, considering these insights in the corresponding cell-tissue experiments. A deeper insight in the correlation between quantum theory and bio-macromolecular systems has a tremendous impact on the understanding of numerous processes in live. In this publication we have provided introduced beside our DFT results also a suited cellular test system where these theoretical data can be tested on a cellular level.

## Data Availability

The original contributions presented in the study are included in the article/Supplementary Materials, further inquiries can be directed to the corresponding author/s.
